# p2rx3 Knockout Mice Have Altered Energy Metabolism in Hippocampal Neurons

**DOI:** 10.32607/actanaturae.27551

**Published:** 2025

**Authors:** A. S. Zelentsova, M. V. Pokrovskii, E. A. Patrakhanov, V. S. Shmigerova, M. Yu. Skorkina, A. V. Deykin

**Affiliations:** Belgorod State National Research University, Belgorod, 308015 Russia

**Keywords:** p2rx3 gene, hippocampus, primary mixed neuronal culture, mitochondrial respiration

## Abstract

The hippocampus is a key component of the brain that is associated with the formation of longterm memory, the energy metabolism of neurons playing a pivotal role in its mechanisms. The P2X3 receptor in the hippocampus is considered an attractive target when searching for novel biologically active substances that could work to reduce anxiety, epileptic conditions, and improve cognitive functions. In this work, the intensity of mitochondrial respiration, the glycolytic capacity, and the energy phenotype of hippocampal neurons were studied in *p2rx3 *knockout mice. The *p2rx3 *knockout mice were engineered by genome editing using the CRISPR/Cas9 system. The primary mixed culture of hippocampal neurons was derived from two-day-old newborn mice with the *p2rx3^-/-^*and *p2rx3^+/-^*genotypes. Mitochondrial respiration was measured on a Seahorse Bioscience HS mini Cell Metabolism Analyzer (Agilent, USA) using the appropriate kits for the Mitostress test, glycotest, and energy phenotype assessment test. The transgenic mice with the *p2rx3^-/-^* genotype were characterized by an aerobic type of mitochondrial respiration, an increase in ATP production by 84.4% (*p* < 0.05), an increase in maximum respiration by 72.3% (*p* < 0.05), and a 36% (*p* < 0.05) increase in the respiratory reserve. Meanwhile, the spare respiratory capacity of mitochondria, the rate of glycolysis, and the glycolytic capacity in these mice were reduced by 36.6, 75.7 and 78.6% (*p* < 0.05), respectively. Our findings indicate that mitochondria work at close to maximum energy capacity. The *p2rx3 *knockout animals are a unique model for the search for pharmacological targets that can help correct the energy metabolism of brain cells and eliminate cognitive dysfunctions.

## INTRODUCTION

Energy metabolism of hippocampal neurons is closely related to cognitive functions, memory, and learning processes [1]. The mechanisms of synaptic signal transmission in the hippocampus involve the ATP molecule and purine receptors [2]. The functional attribute of the P2X receptor family is to generate intracellular Ca^2+^ signals when the membrane potential is close to its physiological resting level [3]. The P2X3 receptor in the hippocampus is an attractive target for the study of anxiety and motivation processes [4], as well as that of the pathogenesis of epileptic states [5]. Abnormalities in hippocampal synaptic plasticity, as well as impaired long-term depression in the CA1 and CA3 synapses and the dentate gyrus of the hippocampus, were observed in mice lacking the P2X3 receptor (P2X3KO). Yet P2X3KO mice still performed adequately on spatial learning tests in a water maze, suggesting that knocking out the *p2rx3* gene improved learning. In addition, P2X3KO mice performed better in a task that involved visually locating and swimming to a platform compared to wildtype mice [5]. Despite the numerous studies that have been devoted to various aspects of how the P2X3 receptor functions [6], the question of the relationship between the receptor and mitochondrial function that determines the activity of cellular metabolism, calcium homeostasis, and, as a consequence, the regulation of the synaptic plasticity of the hippocampus in the central nervous system remains poorly studied. Our study focuses on the intensity of mitochondrial respiration and glycolytic capacity. It also assesses the energy phenotype of hippocampal neurons in *p2rx3* knockout mice.

## EXPERIMENTAL PART

**Work with laboratory animals**

The animals were kept in a conventional vivarium of the Belgorod State National Research University, with artificially regulated daylight hours (12 h dark and 12 h light), under a temperature regime of 22–26°C. They had free access to food and water. The work was guided by the ethical principles that regulate the handling of laboratory animals in accordance with the European Convention for the Protection of Vertebrate Animals Used for Experimental and other Scientific Purposes (ETS No. 170). All painful manipulations with the animals were performed in accordance with regulatory standards: Directive 2010/63/EU of the European Parliament and of the Council of the European Union of September 22, 2010 on the protection of animals used for scientific purposes and approved by the Committee for the Control of the Care and Use of Laboratory Animals of Belgorod National Research University (expert opinion No. 01i/23 dated January 23, 2023).

**Fig. 1:**
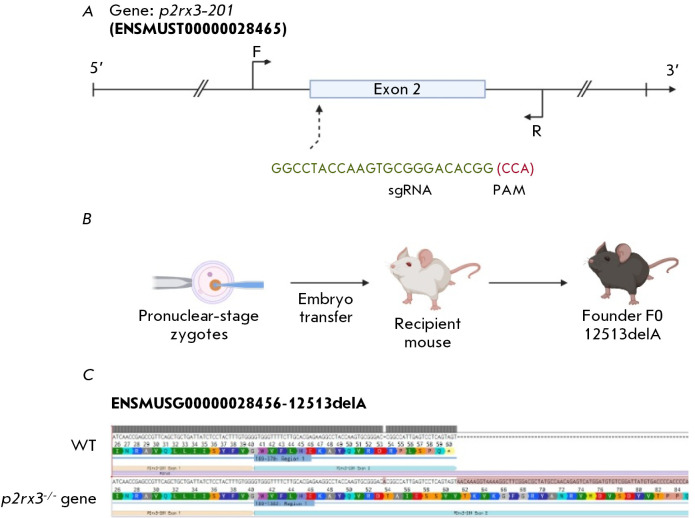
Creating a *p2rx3 *knockout mouse. (*A*) Guide RNA selected for gene editing using the CRISPR/Cas9 method. (*B*) The process of pronuclear injection and creation of the F0 generation. (*C*) The transcript of the *p2rx3 *gene from a wild-type (WT) mouse and a mouse with a knockout of this gene

The animals with an edited genome were obtained by microinjection of the genetic construct into the pronucleus of the donor mouse zygote, followed by transplantation of the reproductive material into the recipient’s female (*Fig. 1B*) [7, 8, 9]. A single guide RNA (sgRNA) recognizing the sequence of the second exon of the *p2rx3 *gene was selected using the CHOPCHOP online search tool. The selected sgRNA 5’-GGCCTACCAAGTGCGGGACACGG(CCA)-3’ (PAM, shown in parentheses) (*Fig. 1A*), which had no off-target sites with fewer than three mismatches, was tested in a series of control experiments on blastocysts and used to generate *p2rx3 *knockout mice.

**Genetic analysis of the offspring**

Genotyping of mice was performed by Sanger sequencing of the PCR product containing the recognition zone of the selected sgRNA (forward and reverse primers 5’-ACTAAGCAGGAACTCATCCCAA-3’ and 5’-CATAATCCGACACATCCATGAC-3’) (Eurogen, Russia), *Fig. 1A*, in the Genome Collective Use Center according to the recommended protocol. The results were analyzed using the Decodr Internet resource.

Animals with a frameshift in the *p2rx3* gene by one nucleotide in exon 2 were obtained, leading to the replacement of six codons and formation of a stop codon (*Fig. 1C*). The mutation was successfully fixed in the first generation. Mice with homo and heterozygous mutation are viable. The animals were transferred to the C57Bl/6 genetic background.

**Isolation, seeding, and cultivation of a primary mixed culture of the hippocampus of newborn mice**

For the purpose of performing metabolic tests, two groups of animals, with the *p2rx3^-/-^*(experimental) and *p2rx3^+/-^*(control) genotypes, were formed, each group containing 30 animals. The primary mixed culture of hippocampal neurons was obtained from 2-day-old newborn mice. Mice were euthanized by cervical dislocation. The skin was cut with scissors along the line of the skull base. The head was separated and placed in a tray with crushed ice. The brain was removed and placed in a Petri dish with chilled phosphate-buffered saline (PBS, pH 7.4). The cerebral hemispheres were separated under a binocular microscope (Leica, Germany). The hippocampus was placed on a glass slide with a “well” in a drop of cooled phosphate-buffered saline (PBS, pH 7.4), divided into six to eight pieces, and transferred into a test tube with 0.25% trypsin–EDTA. The tissue was trypsinized in a 0.25% trypsin–EDTA solution (Gibco, 25200056) in a Binder incubator (Germany) at an atmosphere of 5% CO_2_ for 20 min at 37°C. After trypsinization, the cell suspension was washed three times in PBS (pH 7.4). A total of 2 mL of a neurobasal medium (Gibco, 21103049) containing a 2% B-27 protein supplement (Gibco, 17504044), 0.5 mM L-glutamax (Gibco, 25030081), and 1% PenStrep (PanEco, Russia) was added to the resulting suspension [10].

**Metabolic tests**

Two groups of animals (30 per group) with the* p2rx3^-/-^*(experimental) and *p2rx3^+/-^*(control) genotypes were formed to conduct metabolic tests.

***Evaluation of mitochondrial respiration parameters.***

Mitochondrial respiration was measured on a Seahorse Bioscience HS mini Cell Metabolism Analyzer (Agilent). A sensor cartridge (Agilent) was hydrated 24 h prior to analysis by filling it with a calibration standard solution (Seahorse XF Calibrant) (200 μL in each well). The cartridge was placed in an incubator without CO_2_ at 37°C for 24 h. The assay medium was prepared using Seahorse XF DMEM Media containing glucose at a final concentration of 10 mM, pyruvate 1 mM, and *L*-glutamine 2 mM, according to the manufacturer’s recommendations. The MitostressTest kit was used to assess the function of mitochondria. Stock solutions were prepared according to the manufacturer’s instructions. The kit includes oligomycin, FCCP, and a mixture of rotenone and antimycin A. In the experiment, working solutions were prepared to the following final concentrations per well: oligomycin, 1 μM; FCCP, 2.5 μM; and rotenone/antimycin A, 0.5 μM. Mitostressors were injected into the cell cultures through the ports of the Agilent Seahorse XFp sensor cartridge (Agilent, USA). The cartridge was calibrated; the calibration plate was then replaced with a plate with the cells, and measurements of the rate of oxygen consumption (OCR) indicating the degree of mitochondrial respiration in a cell were conducted. Three technical measurements were performed in each experimental and control cell. Data were normalized by the number of cells. The basal respiration, proton leak, maximum respiration, spare respiratory capacity, non-mitochondrial respiration, ATP production, and the respiratory coupling coefficient were calculated using the Multi- File Seahorse XF Mitostress test software product (USA). Non-mitochondrial respiration was taken as the minimum measured OCR value after injection of the rotenone/antimycin A mixture. Basal respiration was calculated as the last measured OCR value before the first injection minus nonmitochondrial respiration. Maximum respiration was evaluated as the difference between the maximum OCR values after FCCP injection and non mitochondrial respiration. Proton loss was calculated as the minimum OCR value after oligomycin injection minus nonmitochondrial respiration. ATP production was counted after oligomycin addition as the difference between the last OCR value measured before oligomycin injection and the minimum OCR value after oligomycin injection. The spare respiratory capacity of mitochondria was determined as the difference between maximal respiration and basal respiration. The respiratory efficiency coefficient was measured as the ratio between ATP production and basal respiration.

***Studying the cellular bioenergetic balance.***

The energy phenotype of neurons was assessed using a Cell Energy Phenotype kit (Kit 103325-100, Agilent). The kit contained oligomycin (ATP synthase inhibitor) at a final concentration of 100 μM and FCCP (mitochondrial uncoupler) at a final concentration of 100 μM. The concentration of the oligomycin/FCCP stress solution added to the cartridge port was 1.0/1.0 μM. Based on the results of measurements using the Multi-File Seahorse XF Cell Energy Phenotype software (USA), stress OCR and ECAR were calculated according to the formulas:

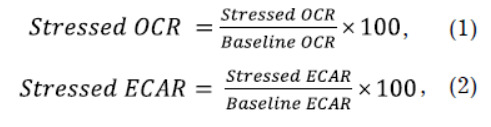



where *Stressed OCR *is the stress phenotype by the rate of oxygen uptake, %;

*Stressed ECAR *is the stress phenotype by the rate of medium acidification, %;

*Stressed OCR/ECAR *is the rate of oxygen uptake / rate of medium acidification after the addition of mitostressors (a mixture of oligomycin and FCCP) to the medium;

*Baseline OCR/ECAR *is the rate of oxygen uptake / rate of medium acidification before the addition of stressors.

**Glycotest stress.**

The analysis was performed using the Glycolysis Stress Test kit.

The kit used glucose at a final concentration of 10 mM, oligomycin (1 μM), and 2-deoxyglucose (500 mM), which were injected into cell cultures through the ports of the Agilent sensor cartridge Cartridge (Seahorse XFp). The cartridge was calibrated, the calibration plate was then replaced with a plate with cells, and the extracellular acidification rate of the medium (ECAR) was measured. The data were normalized by the number of cells. The glycolysis rate, glycolytic capacity of neurons, glycolytic reserve, and non-glycolytic population were calculated using the Multi-File Seahorse XF Glycotest software (USA). The glycolysis rate was calculated as the difference between the maximum ECAR value before oligomycin injection and the last ECAR measurement before glucose injection. The glycolytic capacity was calculated as the difference between the maximum measured ECAR after oligomycin injection and the last ECAR measurement before glucose injection. The glycolytic reserve was estimated as the glycolytic capacity divided by the glycolysis rate (mpH/pmol/min/cell) and multiplied by 100%. Nonglycolytic acidification was taken into account as the last ECAR measurement before glucose injection. All metabolic tests were performed in quadruplicates with three technical measurements each.

**Statistical analysis**

The experimental data were processed using the Wave 2.6 software (USA) and the Excel 10.0 descriptive statistics package. The experimental data are presented as the median and standard deviation (M ± SD). Considering that all the obtained numerical data do not obey the normal distribution hypothesis, the statistical significance of the results was assessed using the Mann–Whitney U test for samples with the number of measurements *n *≤ 20. The critical level of significance was considered at *p* = 0.05.

## RESULTS

**Fig. 2:**
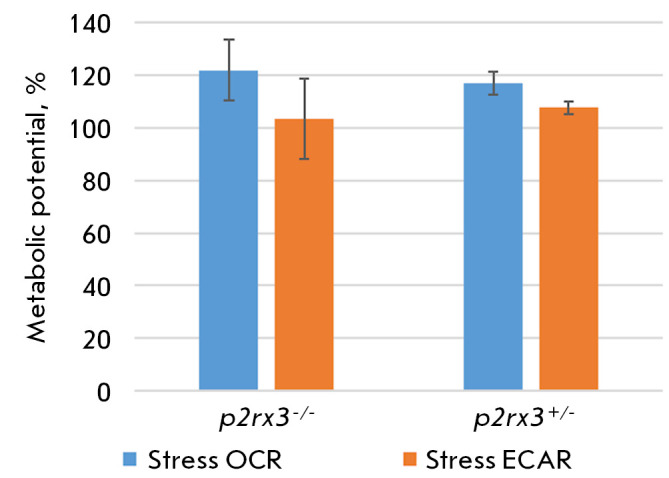
The metabolic potential of the primary mixed culture of hippocampal neurons of transgenic *p2rx3 *knockout mice

The energy phenotype of a primary mixed culture of hippocampal neurons was studied in transgenic animals. In tests of this type, the rate of extracellular acidification (ECAR) is a reliable indicator of the glycolysis rate. However, when highly aerobic cells are exposed to stress, carbon dioxide production by mitochondria can provoke a rise in ECAR [11] and increase the contribution of glycolysis to the metabolic potential. The susceptibility of the hippocampal cells of the engineered transgenic mice to this effect was assessed using this test. The hippocampal neurons of both the homozygous and heterozygous transgenic animals were not susceptible to this effect. The energy phenotype of mitochondrial respiration of the primary mixed culture of hippocampal neurons of transgenic animals is aerobic respiration relying on oxidative phosphorylation. In the primary mixed culture of hippocampal neurons obtained from *p2rx3^-/-^*mice, the ratio of the oxygen consumption rate (OCR) to the extracellular acidification rate (ECAR) was 1 : 2; in mice with the *p2rx3^+/-^*genotype, *OCR/ECAR *= 1.1. No clearly expressed differences in the metabolic phenotype of hippocampal neurons were revealed between the studied groups of animals (*Fig. 2*).

When studying the features of mitochondrial respiration, the real-time curves of the oxygen uptake rate were recorded (*Supplementary Fig. S1A*). An increase in almost all the parameters of mitochondrial respiration was denoted, except for the spare respiratory capacity obtained from *p2rx3^-/-^*homozygotes (*Table 1*).

**Table 1:** Parameters of the mitochondrial respiration of hippocampal neuron cultures from transgenic p2rx3 knockout mice

Parameters	р2rx3^+/-^ (control)	р2rx3^-/-^ (experiment)	P U test ≤ 17
Nonmitochondrial respiration, pmol/min/cell	27.1 ± 5.3	56.9 ± 18.0^*^	0
Basal respiration, pmol/min/cell	65.9 ± 12.2	238.1 ± 7.9^*^	0
Maximal respiration, pmol/min/cell	124.3 ± 8.9	320.9 ± 18.5^*^	0
Proton (H+) loss, pmol/min/cell	14.5 ± 8.1	40.4 ± 16.4	19
ATP production, pmol/min/cell	36.1 ± 12.8	231.5 ± 9.9^*^	0
Respiratory reserve, pmol/min/cel	l 56.0 ± 16.4	87.4 ± 14.3^*^	0
Spare respiratory capacity, %	188.3 ± 45.20	137.8 ± 7.2^*^	0
Respiratory efficiency coefficient, %	58.8 ± 21.6	103.4 ± 10.4^*^	1

^*^*Significance of differences at p < 0.05 compared to the control according to the Mann–Whitney U test.

According to the data in Table 1, non mitochondrial, basal and maximal respiration, as well as the respiratory reserve, significantly increased in the culture of neurons obtained from *p2rx3*^-/-^ mice by 52.4% (*p* < 0.05), 72.3% (*p* < 0.05), and 61.3% (*p* < 0.05), respectively, compared to the control. The culture of hippocampal neurons collected from mice was characterized by an increase in the ATP production by 84.4% (*p* < 0.05); respiratory reserve, by 36% (*p * < 0.05); and respiratory efficiency coefficient, by 43% (*p* < 0.05) compared to the control. Due to the high intensity of mitochondrial respiration, the reserve respiratory capacity of the primary mixed culture of the hippocampus of *p2rx3*^-/-^ mice was down by 36.6% (*p* < 0.05) compared to the control.

**Fig. 3:**
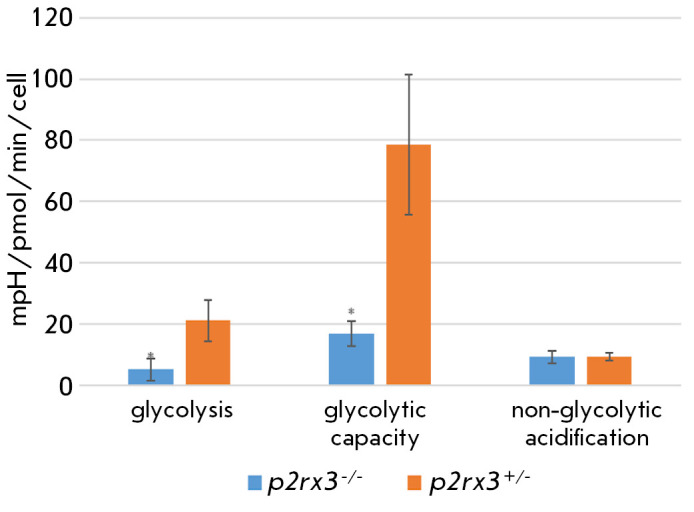
Glycolytic function indices of the primary mixed culture of hippocampal neurons from *p2rx3 *knockout mice. * – significance of differences at *p* < 0.05 compared to heterozygotes according to the Mann–Whitney U test

In the primary mixed culture of hippocampal neurons from *p2rx3^-/-^*mice, the glycolysis rate and glycolytic capacity were significantly reduced, by 75.7% (*p* < 0.05) and 78.6% (*p* < 0.05) compared to similar indicators in mice with the *p2rx3^+/-^*genotype (*Fig. 3*).

The glycolytic reserve of hippocampal neurons of* p2rx3^-/-^*mice increased almost two-fold and amounted to 351.3 ± 158.2% compared to that of the hippocampal culture of *p2rx3^+/-^*mice (163.2 ± 60.5%). The realtime glycolytic curves are presented in *Supplementary Fig. S1B.*

## DISCUSSION

The primary mixed culture of hippocampal neurons obtained from animals of both the *p2rx3^-/-^*and* p2rx3^+/-^*genotypes is characterized by aerobic respiration when the cell uses predominantly oxidative phosphorylation. The experimentally determined aerobic type of metabolism of hippocampal neurons and the absence of any switch of the energy phenotype in response to the introduction of mitochondrial respiration stressors are indicative of the enhanced bioenergetic function of mitochondria.

We found that hippocampal neurons from *p2rx3^-/-^* knockout mice have increased basal and nonmitochondrial respiration compared to hippocampal neurons derived from *p2rx3^+/-^*mice. Our findings also show that *p2rx3^-/-^*hippocampal neurons exhibit high ATP production and a reduced glycolysis rate and glycolytic capacity, which essentially characterizes maximal capacity to generate ATP during glycolysis. These findings indicate that *p2rx3^-/-^*knockout hippocampal neurons in the basal state already operate close to the peak of their mitochondrial energy capacity. The combination of intense mitochondrial respiration, together with the leak of protons, which further produce reactive oxygen species (ROS), and together with reduced spare respiratory capacity, may cause difficulties for such neurons to cope with significant fluctuations in bioenergetic needs during various cellular stresses, as well as during aging. Increased oxidative phosphorylation in neurons is the main cause of elevated ROS levels [12]. The main question is why *p2rx3 *knockout neurons have such high energy requirements. According to the published data, the dopamine neurons of mice with Alzheimer’s disease have higher energy requirements [13]. Such energy expenditure is associated with large axonal arborization, which requires highly efficient production of mitochondrial ATP because of the increased mitochondrial density that characterizes these terminals [13]. In our study, we did not determine the degree of neuronal branching in the engineered transgenic mice and can only hypothesize about the role played by ionotropic receptors in enhancing the bioenergetics of hippocampal neurons based on the data available in the literature.

The *p2rx3 *gene encodes the P2X3 receptor, which is expressed in various brain regions, including pyramidal neurons, dentate granule cells, and hippocampal interneurons [14]. Considering that the hippocampus is a key structure of the brain associated with the formation of long-term memory, changes in the activity of ionotropic receptors — purinergic (P2X) and glutamatergic (NMDA) — can modulate plasticity and hippocampus-dependent learning, as well as memory. This plasticity is based on the activation of hippocampal kinases and changes in the intracellular calcium levels [15]. There are publications that describe cross-interactions of P2X receptors with NMDA. It has been proved that activation of P2X receptors inhibits Ca^2+^ currents through NMDA receptors. The functional significance of such interaction is related to the fact that P2X receptors act as low-frequency filters of the calcium signal under physiological rest conditions when membrane depolarization is not required for calcium entry as is the case with NMDA receptors [3]. The *p2rx3 *knockout mice demonstrated impaired long-term depression in the hippocampal CA1, CA3, and dentate granule cell synapses, as well as improved learning and spatial orientation [5]. Inhibition of P2X family receptors (P2X3, P2X4, and P2X6 families) enhances the induction of long-term memory [3]. It is known that during the long-term potentiation underlying memory and learning, mitochondrial energy production is altered [16], the activity of the mitochondrial calcium pump increases [17], and the expression of mitochondrial genes is enhanced [18]. Blocking mitochondrial oxidative phosphorylation results in significant impairment of long-term potentiation [19]. Mitochondrial energy production is critical for transmitter release via vesicle exocytosis, mobilization of synaptic reserve pool vesicles, and regulation of synaptic strength [20]. The increased bioenergetic needs of neurons may be associated with excessive activation of NMDA receptors. According to researchers, activation of NMDA receptors increases the volume of spines in cultured hippocampal neurons and increases the surface expression of AMPA receptors [21]. Regulation of dendritic division of mitochondria, accompanied by an increase in the Ca^2+^ content in the mitochondrial matrix, and mediation by activation of Ca^2+^ and calmodulin-dependent protein kinase II (CAMK II) has been described [22]. One of the most recent studies has shown the localization of an NMDA-like receptor on the mitochondrial membrane, which enhances the activity of ATP synthase and ATP production by neurons [23].

## CONCLUSION

Our findings indicate that *p2rx3 *gene knockout mice have mitochondria with increased bioenergetic function. Transgenic mice with the *p2rx3-/- *genotype are characterized by an aerobic type of mitochondrial respiration, high ATP production, increased basal and non mitochondrial respiration, increased neuron loss, a fairly high level of the respiratory efficiency coefficient, while the spare respiratory capacity of mitochondria, the glycolysis rate, and glycolytic capacity are reduced. The data obtained indicate that mitochondria work close to the peak of their energy capacity. It is possible that such activation of the cellular energy metabolism is associated with reciprocal interactions between ionotropic purinergic and glutamatergic receptors. An important and open question remains: how will the bioenergetic balance of hippocampal neurons change in *p2rx3 *knockout animals in response to blockade of the NMDA receptor? Understanding the interactions between purinergic and glutamatergic receptors is important, since these receptors are involved in certain types of hippocampus- dependent memories. The *p2rx3 *knockout animals in this study are a unique model for searching for pharmacological targets in efforts to correct the energy metabolism of brain cells and eliminate cognitive dysfunctions.

## Abbreviations

ROS: reactive oxygen species
CNS: central nervous system
EDTA: ethylenediaminetetraacetic acid
AMPA: alpha-amino-3-hydroxy-5-methyl-4-isoxazolepropionic acid
ATP: adenosine triphosphate
CAMK II: calcium calmodulin-dependent protein kinase
CRISPR/Cas9: clustered regularly interspaced short palindromic repeats
ECAR: extracellular acidification rate
FCCP: carbonyl cyanide-4 (trifluoromethoxy) phenylhydrazone
NMDA: ionotropic glutamate receptor selectively binding N-methyl- D-aspartate
OCR: oxygen consumption rate
PBS: phosphate buffered saline
P2X3KO: mice lacking the P2X3 receptor

## References

[1] Rubin R.D., Watson P.D., Duff M.C., Cohen N.J. (2014). Front. Hum. Neurosci..

[2] Lalo U., Pankratov Y. (2023). Neuropharmacology..

[3] Pankratov Y.V., Lalo U.V., Krishtal O.A. (2002). J. Neurosci..

[4] Wang Y., Mackes J., Chan S., Haughey N.J., Guo Z., Ouyang X., Furukawa K., Ingram D.K., Mattson M.P. (2006). J. Neurochem..

[5] Zhou X., Ma L.M., Xiong Y., Huang H., Yuan J.X., Li R.H., Li J.N., Chen Y.M. (2016). Neurochem. Res..

[6] Oparin P., Khokhlova O., Cherkashin A., Nadezhdin K., Palikov V., Palikova Y., Korolkova Y., Mosharova I., Rogachevskaja O., Baranov M. (2025). Molecular Therapy.

[7] Gurskiy Y.G., Garbuz D.G., Soshnikova N.V., Krasnov A.N., Deikin A., Lazarev V.F., Sverchinskyi D., Margulis B.A., Zatsepina O.G., Karpov V.L. (2016). Cell Stress Chaperones..

[8] Silaeva Y.Y., Kirikovich Y.K., Skuratovskaya L.N., Deikin A.V. (2018). Russ. J. Develop. Biol..

[9] Kalmykov V.A., Kusov P.A., Yablonskaia M.I., Korshunov E.N., Korshunova D.S., Kubekina M.V., Silaeva Y.Yu., Deykin A.V., Lukyanov N.E. (2018). Research Results in Pharmacology..

[10] Zelentsova A.S., Borisova A.Y., Shmigerova V.S., Skorkina M.Y., Deykin A.V. (2024). Genes and Cells..

[11] Vohwinkel C.U., Lecuona E., Sun H., Sommer N., Vadász I., Chandel N.S., Sznajder J.I. (2011). J. Biol. Chem..

[12] Hill B.G., Benavides G.A., Lancaster J.R. Jr., Ballinger S., Dell'Italia L., Jianhua Z., Darley-Usmar V.M. (2012). Biol. Chem..

[13] Pacelli C., Giguère N., Bourque M.J., Lévesque M., Slack R.S., Trudeau L.É. (2015). Curr. Biol..

[14] Jang I.S., Rhee J.S., Kubota H., Akaike N., Akaike N. (2001). J. Physiol..

[15] Medina J.H., Viola H. (2018). Front. Mol. Neurosci..

[16] Wieraszko A. (1982). Brain Res..

[17] Stanton P.K., Schanne F.A. (1986). Brain Res..

[18] Williams J.M., Thompson V.L., Mason-Parker S.E., Abraham W.C., Tate W.P. (1998). Brain Res. Mol. Brain Res..

[19] Cunha R.A., Vizi E.S., Ribeiro J.A., Sebastião A.M. (1996). J. Neurochem..

[20] Ivannikov M.V., Sugimori M., Llinás R.R. (2013). J. Mol. Neurosci..

[21] Divakaruni S.S., van Dyke A.M., Chandra R., LeGates T.A., Contreras M., Dharmasri P.A., Higgs H.N., Lobo M.K., Thompson S.M., Blanpied T.A. (2018). Neuron..

[22] Duarte F.V., Ciampi D., Duarte C.B. (2023). Cell Mol. Life Sci..

[23] Korde A.S., Maragos W.F. (2021). Mitochondrion..

